# Dissincronia Ecocardiográfica de Estresse e Strain Longitudinal Miocárdico Ventricular em Pacientes com Cardiomiopatia Chagásica: Fenótipos Prognósticos Além da Fração de Ejeção

**DOI:** 10.36660/abc.20260631

**Published:** 2026-11-04

**Authors:** Marcelo Luiz Campos Vieira, Bruno Pereira Valdigem, Edgar Daminello

**Affiliations:** 1 Hospital Israelita Albert Einstein São Paulo SP Brasil Hospital Israelita Albert Einstein, São Paulo, SP – Brasil; 2 Hospital das Clínicas da Faculdade de Medicina da Universidade de São Paulo Instituto do Coração São Paulo SP Brasil Instituto do Coração do Hospital das Clínicas da Faculdade de Medicina da Universidade de São Paulo, São Paulo, SP – Brasil; 3 Instituto Dante Pazzanese de Cardiologia São Paulo SP Brasil Instituto Dante Pazzanese de Cardiologia, São Paulo, SP – Brasil

**Keywords:** Ecocardiografia sob Estresse, Doença de Chagas, Cardiomiopatia Chagásica

O estudo da deformação miocárdica do ventrículo esquerdo (VE) (*Strain*) é uma modalidade ecocardiográfica capaz de identificar disfunção sistólica mesmo antes da queda na fração de ejeção (FE).^1–3^ O *strain* pode ser avaliado nos planos longitudinal, radial e circunferencial, bem como em suas combinações, fornecendo novos parâmetros fisiológicos cardíacos, como *twist*, torção e *strain* de área.

O *strain* do eixo longitudinal do VE é o parâmetro de deformação cardíaca com as evidências científicas mais robustas e o uso mais relevante na prática clínica.^1–3^ A terapia de ressincronização cardíaca (TRC) é uma opção terapêutica já consolidada em diretrizes internacionais e promove uma redução significativa na morbidade e mortalidade, como demonstrado por estudos marcantes como MADIT-CRT e CARE-HF.^2,4,5^ É recomendada como tratamento de classe I para pacientes com cardiomiopatia dilatada, sintomática, sob tratamento clínico otimizado, eletrocardiograma com padrão de bloqueio de ramo esquerdo (BRE), duração do QRS ≥ 150 ms e fração de ejeção do VE abaixo de 35%.^2^

A análise da dissincronia cardíaca pode ser realizada utilizando múltiplas técnicas ecocardiográficas, incluindo modo M, Doppler convencional, Doppler tecidual, ecocardiografia tridimensional e até mesmo análise de deformação ventricular.^1–3,6–9^ A observação de padrões específicos de movimento e mecânica cardíaca, como o balanço apical e o flash septal, tem sido descrita como parâmetros importantes para determinar uma resposta positiva à TRC em pacientes com insuficiência cardíaca avançada.^10,11^

Entretanto, pacientes sem padrão de BRE não apresentaram um benefício claro, apesar das diretrizes publicadas recomendarem o procedimento para todos os pacientes com intervalo QRS de 150 ms ou maior, sintomas refratários e terapia médica otimizada.^10^

A taxa de resposta à TRC pode ser melhorada com o uso da ecocardiografia, com análise da dissincronia SLAP do VE nos eixos longitudinal e radial. A análise radial pode revelar a diferença de tempo entre a deformação radial máxima dos segmentos médios das paredes anterosseptal e inferolateral. Uma medida superior a 130 ms caracteriza pacientes com maior taxa de resposta. Além da dissincronia radial, a identificação de um padrão de estiramento septal de rebote é um preditor independente. Esse padrão reflete a incoordenação da contração cardíaca com consequente redução do desempenho miocárdico.

A inclusão da dissincronia mecânica como critério de seleção para TRC aumentou as taxas de resposta de 50% para 75% em pacientes sem BRE e com duração do QRS ≥150 ms, e de 38% para 62% naqueles com duração do QRS entre 130 e 149 ms. Dado que dois terços ou mais dos pacientes com doença cardíaca chagásica crônica apresentam bloqueio de ramo direito — associado ou não a bloqueio fascicular esquerdo — qualquer evidência que apoie intervenções nessa população é muito bem-vinda.

Um aspecto importante é que a presença de reserva contrátil é um marcador reconhecido de melhor prognóstico com a TRC; além disso, um aumento na dissincronia intraventricular após o estresse está associado ao agravamento da regurgitação da válvula mitral e a alterações no débito cardíaco, independentemente do consumo máximo de oxigênio durante o teste cardiopulmonar de exercício.

Assim, a análise combinada da dissincronia mecânica, determinada pela observação do *strain* ventricular e da reserva contrátil durante o estresse cardíaco em pacientes com cardiomiopatia chagásica, pode fornecer novos insights sobre a fisiologia cardíaca e a potencial indicação para TRC. Esse foi o objetivo de Athayde GAT et al. em um estudo publicado nos Arquivos Brasileiros de Cardiologia, envolvendo 40 pacientes com cardiomiopatia chagásica submetidos à análise da dissincronia cardíaca (método de Yu), *strain* longitudinal do VE e reserva miocárdica durante teste de estresse físico.^12^ O acompanhamento clínico médio foi de 729 dias para a observação do desfecho primário combinado de morte, transplante cardíaco ou hospitalização por insuficiência cardíaca.^12^ Os autores observaram que "Pacientes sem reserva contrátil e sem melhora da dissincronia durante o exercício apresentaram sobrevida livre de eventos significativamente menor em comparação com os demais grupos".^12^ Esses autores concluíram que a "Integração da reserva contrátil derivada do *strain* e da dissincronia intraventricular durante o estresse fisiológico pode ajudar a identificar fenótipos prognósticos além da fração de ejeção na cardiomiopatia chagásica crônica e apoia o conceito de fenotipagem mecânica dinâmica na cardiomiopatia avançada".^12^

Não há dúvida de que a análise ecocardiográfica combinada do strain biventricular e da reserva contrátil, avaliada em repouso e durante o estresse, constitui uma investigação necessária com alto potencial diagnóstico para um melhor conhecimento da fisiopatologia da cardiomiopatia chagásica.

Portanto, gostaríamos de parabenizar profundamente os autores pela excelência de sua pesquisa, que sem dúvida avança nossa compreensão da cardiomiopatia da doença de Chagas - uma condição que continua sendo uma grande preocupação de saúde pública em nosso país e na América Latina, dado o número significativo de pacientes afetados pela doença.^13–15^

## References

[1] Almeida ALC, Melo MDT, Bihan DCSL, Vieira MLC, Pena JLB, Del Castillo JM (2023). Position Statement on the Use of Myocardial Strain in Cardiology Routines by the Brazilian Society of Cardiology's Department Of Cardiovascular Imaging - 2023. Arq Bras Cardiol.

[2] Thomas JD, Edvardsen T, Abraham T, Appadurai V, Badano L, Banchs J (2025). Clinical Applications of Strain Echocardiography: A Clinical Consensus Statement from the American Society of Echocardiography Developed in Collaboration with the European Association of Cardiovascular Imaging of the European Society of Cardiology. J Am Soc Echocardiogr.

[3] Hotta VT, Abduch MCD, Vieira MLC, Vilela AA, Bocchi EA (2022). Three and Two-Dimensional Cardiac Mechanics by Speckle Tracking are Predictors of Outcomes in Chagas Heart Disease. Sci Rep.

[4] Moss AJ, Hall WJ, Cannom DS, Klein H, Brown MW, Daubert JP (2009). Cardiac-Resynchronization Therapy for the Prevention of Heart-Failure Events. N Engl J Med.

[5] Cleland JG, Daubert JC, Erdmann E, Freemantle N, Gras D, Kappenberger L (2005). The Effect of Cardiac Resynchronization on Morbidity and Mortality in Heart Failure. N Engl J Med.

[6] Yu CM, Chau E, Sanderson JE, Fan K, Tang MO, Fung WH (2002). Tissue Doppler Echocardiographic Evidence of Reverse Remodeling and Improved Synchronicity by Simultaneously Delaying Regional Contraction after Biventricular Pacing Therapy in Heart Failure. Circulation.

[7] Yu CM, Lin H, Zhang Q, Sanderson JE (2003). High Prevalence of Left Ventricular Systolic and Diastolic Asynchrony in Patients with Congestive Heart Failure and Normal QRS Duration. Heart.

[8] Yu CM, Zhang Q, Fung JW, Chan HC, Chan YS, Yip GW (2005). A Novel Tool to Assess Systolic Asynchrony and Identify Responders of Cardiac Resynchronization Therapy by Tissue Synchronization Imaging. J Am Coll Cardiol.

[9] Suffoletto MS, Dohi K, Cannesson M, Saba S, Gorcsan J (2006). Novel Speckle-Tracking Radial Strain from Routine Black-and-White Echocardiographic Images to Quantify Dyssynchrony and Predict Response to Cardiac Resynchronization Therapy. Circulation.

[10] Glikson M, Nielsen JC, Kronborg MB, Michowitz Y, Auricchio A, Barbash IM (2021). 2021 ESC Guidelines on Cardiac Pacing and Cardiac Resynchronization Therapy. Eur Heart J.

[11] Salden OAE, Vernooy K, van Stipdonk AMW, Cramer MJ, Prinzen FW, Meine M (2020). Strategies to Improve Selection of Patients without Typical Left Bundle Branch Block for Cardiac Resynchronization Therapy. JACC Clin Electrophysiol.

[12] Athayde GAT, Borges BCC, Pinheiro AO, Souza AL, Oliveira AP, Martins SAM (2026). Interações entre Strain e Dissincronia na Ecocardiografia sob Estresse Identificam Fenótipos Prognósticos na Cardiomiopatia Chagásica: Um Estudo Piloto. Arq Bras Cardiol.

[13] Rassi DC, Vieira ML, Arruda AL, Hotta VT, Furtado RG, Rassi DT (2014). Echocardiographic Parameters and Survival in Chagas Heart Disease with Severe Systolic Dysfunction. Arq Bras Cardiol.

[14] Rassi DD, Vieira ML, Furtado RG, Turco FP, Melato LH, Hotta VT (2017). Dobutamine Stress Echocardiography Safety in Chagas Disease Patients. Arq Bras Cardiol.

[15] Acquatella H, Asch FM, Barbosa MM, Barros M, Bern C, Cavalcante JL (2018). Recommendations for Multimodality Cardiac Imaging in Patients with Chagas Disease: A Report from the American Society of Echocardiography in Collaboration with the InterAmerican Association of Echocardiography (ECOSIAC) and the Cardiovascular Imaging Department of the Brazilian Society of Cardiology (DIC-SBC). J Am Soc Echocardiogr.

