# Supplementary material for: Dissincronia Ecocardiográfica de Estresse e Strain Longitudinal Miocárdico Ventricular em Pacientes com Cardiomiopatia Chagásica: Fenótipos Prognósticos Além da Fração de Ejeção
Source: Arq Bras Cardiol. 2026 Nov 4;123(9):e20260631. [Article in Portuguese] doi: 10.36660/abc.20260631 (PMC13754394; doi:10.36660/abc.20260631)
Supplement: Translation

# Translation: Stress Echocardiographic Dyssinchrony, Ventricular Myocardial Longitudinal Strain in Chagas Cardiomyopathy Patients: Prognostic Phenotypes Beyond Ejection Fraction

**Authors:** Marcelo Luiz Campos Vieira, Bruno Pereira Valdigem, Edgar Daminello

**Affiliations:** 1 São Paulo SP Brazil Hospital Israelita Albert Einstein, São Paulo, SP – Brazil; 2 São Paulo SP Brazil Instituto do Coração do Hospital das Clínicas da Faculdade de Medicina da Universidade de São Paulo, São Paulo, SP – Brazil; 3 São Paulo SP Brazil Instituto Dante Pazzanese de Cardiologia, São Paulo, SP – Brazil

**Keywords:** Stress Echocardiography, Chagas Disease, Chagas Cardiomyopathy

Left ventricular (LV) myocardial deformation (Strain) study is an echocardiographic modality capable of identifying systolic dysfunction even before the drop in ejection fraction (EF).^1–3^ Strain can be performed in the longitudinal, radial, and circumferential planes, as well as in their combination, bringing new physiological cardiac parameters such as twist, torsion, and area strain. LV longitudinal axis strain is the cardiac deformation parameter with the most robust scientific evidence and the most relevant use in clinical practice.^1–3^ Cardiac resynchronization therapy (CRT) is a therapeutic option already consolidated in international guidelines and promotes a significant reduction in morbidity and mortality, as demonstrated by landmark studies such as MADIT-CRT and CARE-HF.^2,4,5^ It is recommended as a class I treatment for patients with dilated cardiomyopathy, symptomatic, under optimized clinical treatment, electrocardiogram with left bundle branch block (LBBB) pattern, QRS duration ≥ 150 ms, and LV ejection fraction below 35%.^2^

The analysis of cardiac dyssynchrony can be performed using multiple echocardiographic techniques, including M-mode, conventional Doppler, tissue Doppler, three-dimensional echocardiography, and even ventricular strain analysis.^1-,3,6-9^ The observation of specific patterns of cardiac motion and mechanics, such as apical rocking and septal flash, has been described as important parameters for determining a positive response to CRT in patients with advanced heart failure.^10,11^

However, patients without a LBBB pattern did not show a clear benefit, despite published guidelines recommending the procedure for all patients with a QRS interval of 150 ms or greater, refractory symptoms, and optimized medical therapy.^10^

The response rate to CRT can be improved by using echocardiography, with analysis of LV SLAP dyssynchrony in the longitudinal and radial axes. Radial analysis can reveal the time difference between the maximum radial deformation of the mid-segments of the anteroseptal and inferolateral walls. A measurement greater than 130 ms characterizes patients with a higher response rate. In addition to radial dyssynchrony, the identification of a septal rebound stretch pattern is an independent predictor. This pattern reflects incoordination of cardiac contraction with consequent reduction in myocardial performance.

The inclusion of mechanical dyssynchrony as a selection criterion for CRT increased response rates from 50% to 75% in patients without LBBB and a QRS duration ≥150 ms, and from 38% to 62% in those with a QRS duration between 130 and 149 ms. Given that two-thirds or more of patients with chronic Chagasic heart disease present with right bundle branch block—whether or not associated with left fascicular block—any evidence supporting interventions in this population is highly welcome.

An important aspect is that the presence of contractile reserve is a recognized marker of a better prognosis with CRT; furthermore, an increase in intraventricular dyssynchrony following stress is associated with worsening mitral valve regurgitation and changes in cardiac output, independent of peak oxygen consumption during cardiopulmonary exercise testing.

Thus, the combined analysis of mechanical dyssynchrony determined by observing ventricular strain and contractile reserve, observed during cardiac stress in patients with Chagasic cardiomyopathy, may provide new insights into cardiac physiology and the potential indication for CRT. This was the aim of Athayde GAT et al. in a study published in *Arquivos Brasileiros de Cardiologia* involving 40 patients with Chagasic cardiomyopathy who underwent analysis of cardiac dyssynchrony (Yu method), left ventricular longitudinal strain, and myocardial reserve during physical stress testing^12^ The mean clinical follow-up was 729 days for the observation of the combined primary endpoint of death, heart transplantation, or hospitalization for heart failure.^12^ The authors observed that "Patients without contractile reserve and without dyssynchrony improvement during exercise had significantly lower event-free survival compared with the remaining groups".^12^ These authors concluded that the "Integration of strain-derived contractile reserve and intraventricular dyssynchrony during physiologic stress may help identify prognostic phenotypes beyond ejection fraction in chronic Chagas

cardiomyopathy and supports the concept of dynamic mechanical phenotyping in advanced cardiomyopathy".^12^

There is no doubt that the combined echocardiographic analysis of biventricular strain and contractile reserve, assessed at rest and during stress, constitutes a necessary investigation with high diagnostic potential for a better knowledge of the pathophysiology of Chagasic cardiomyopathy.

Therefore, we would like to deeply congratulate the authors on the excellence of their research, which undoubtedly advances our understanding of Chagas disease cardiomyopathy—a condition that remains a major public health concern in our country and in Latin America, given the significant number of patients affected by the disease.^13–15^
